# In Situ Observation of γ-to-α Structural Transformation in Bio-Based Nylon 5,6 Fibers via X-Ray Diffraction and DFT Analysis

**DOI:** 10.3390/polym17172385

**Published:** 2025-08-31

**Authors:** Kukhyun Jo, Hyun Hwi Lee, Sung Hyun Kwon, Hyo Jung Kim

**Affiliations:** 1 Department of Organic Material Science and Engineering, School of Chemical Engineering, Pusan National University, Busan 46241, Republic of Korea; kukhjo@pusan.ac.kr; 2 Institute of Advanced Organic Materials, Pusan National University, Busan 46241, Republic of Korea; 3 Pohang Accelerator Laboratory, POSTECH, Pohang 37673, Republic of Korea; hhleec@postech.ac.kr

**Keywords:** nylon 5,6, phase transformation, γ-to-α transition, X-ray diffraction, DFT, bio-based polymer

## Abstract

This study investigates the structural transformation from the γ-phase into the α-phase in bio-based nylon 5,6 fibers during in situ uniaxial stretching, using X-ray diffraction (XRD) and density functional theory (DFT) calculations. Initially, nylon 5,6 films exhibited a well-defined γ-phase crystalline structure, and the as-spun fibers also retained a γ-phase-dominant structure with partial coexistence of α-phase components. Due to the lattice similarity between the γ- and α-phases, phase separation was challenging in the direction perpendicular to the fiber axis (ab-plane). However, the analysis of the (004) diffraction peaks along the fiber axis (c-axis) enabled the quantitative evaluation of each crystalline component. As the stretching progressed, the α(004) peak intensity gradually increased, indicating a continuous γ-to-α structural transition. Furthermore, DFT calculations revealed that the α-phase has lower energy than the γ-phase, supporting the thermodynamic favorability of the phase transition during elongation. These results provide a comprehensive understanding of the crystalline structure and transformation mechanism in environmentally friendly nylon fibers from both experimental and theoretical perspectives, and offer foundational insights for developing nylon materials with desirable properties through the precise control of crystal phase structures.

## 1. Introduction

### 1.1. Sustainable Bio-Based Polyamide 5,6 (Nylon 5,6)

Polyamide 5,6 (Nylon 5,6) has attracted increasing attention as a sustainable, bio-based polyamide with a lower carbon footprint compared to conventional petroleum-derived nylons. This polymer is synthesized through the polycondensation of 1,5-pentanediamine—produced via microbial fermentation of biomass feedstocks such as corn—and adipic acid. By partially replacing fossil-based monomers with renewable bio-based components, the production process of PA5,6 can significantly reduce greenhouse gas emissions and dependence on non-renewable resources [1]. In fact, commercially available bio-based PA5,6 fibers have been reported to lower CO_2_ emissions by approximately 50% compared to traditional nylon 6 or 6,6 materials [2]. These environmentally friendly attributes align with the global movement toward decarbonization, making Nylon 5,6 a promising low-carbon, high-performance material for applications in textiles, automotive components, and engineering plastics.

### 1.2. Crystalline Structure of Conventional vs. Odd-Methylene Polyamides

From a structural perspective, nylon 5,6 (polyamide 5,6) exhibits distinct crystalline features compared to conventional even–even polyamides such as nylon 6 and nylon 6,6. These conventional polyamides typically crystallize into two polymorphic forms: the α-form and the γ-form. The α-form is a stable monoclinic structure in which all-trans polymer chains form planar hydrogen-bonded sheets, where all CONH groups are aligned in a single direction to establish a regular hydrogen bonding network [3,4].

In contrast, the γ-form appears under conditions of insufficient stretching, rapid quenching, or thermal Brill transitions. This structure features twisted chain conformations, pseudo-hexagonal packing, and less-oriented hydrogen-bonded sheets [5].

However, odd–even polyamides such as nylon 5,6, which contain an odd number of methylene groups, do not follow these conventional crystallization patterns. Due to geometric constraints that prevent all-trans planar packing, PA5,6 forms an “α-like” structure instead of a traditional α-form. Puiggalí et al. reported that in this α-like structure, the amide planes are alternately tilted, resulting in the formation of bifurcated hydrogen bonds [6]. Although this hydrogen bonding arrangement resembles that of the α-form, it is considered a distinct crystalline phase.

Throughout this paper, for simplicity of terminology, we refer to this α-like structure as the “α-phase” of nylon 5,6. Our intention is that, since odd-methylene nylons cannot form the classical α-form and the α-like arrangement represents their only stable and energetically favorable variant, it can reasonably be regarded as the representative α-phase for these systems. Nevertheless, we explicitly clarify that the “α-phase” in this work corresponds to the α-like structure reported in the literature.

The γ-form of nylon 5,6 is also different from the conventional γ-form observed in nylon 6. According to Puiggalí et al., the γ-form of PA5,6 resembles the structure proposed by Kinoshita for nylon 7,7, in which the amide planes are tilted by approximately 30° along the polymer chain, forming a pleated-sheet arrangement [6]. According to Kinoshita, this structure allows for complete hydrogen bonding without relying on pseudo-hexagonal packing and is classified as a relatively stable γ-phase [7].

### 1.3. In Situ Observation of Phase Transition in Nylon 5,6 During Drawing

Despite growing industrial interest in nylon 5,6, systematic studies on its crystalline structure and phase transition behavior remain limited. Morales-Gámez et al. reported the presence of bifurcated hydrogen bonds in solution-grown PA5,6 single crystals and observed a Brill transition from the γ-form to a pseudo-hexagonal structure upon heating [6,8]. While these findings proposed a unique structural model for nylon 5,6, they did not include real-time analysis of structural changes under mechanical stretching.

In this study, we performed in situ observation of the γ → α phase transition in nylon 5,6 fibers during uniaxial deformation using synchrotron-based wide-angle X-ray scattering (WAXS) with high temporal resolution. Moreover, X-ray diffraction (XRD) is a widely established technique for identifying crystalline structures in polymeric materials, and its combination with in situ WAXS has proven to be a particularly powerful approach for monitoring structural evolution in real time [9,10,11,12].

By focusing on the (004) diffraction peak corresponding to the polymer chain direction (c-axis), we quantitatively distinguished the contributions of each crystalline phase and reliably interpreted the orientation and temporal evolution of the structural transition. In addition, we visualized the polymer chain arrangements in the γ- and α-phases using density functional theory (DFT) calculations and quantitatively evaluated the relative stability and energy differences between the two phases. This comprehensive approach encompassing both experimental and theoretical perspectives allows for a deeper insight into the phase transition mechanism of PA5,6 and offers a foundation for the design of sustainable, application-oriented high-performance fibers.

## 2. Materials and Methods

### 2.1. Nylon 5,6 Fiber Samples

The nylon 5,6 polymer used in this study was synthesized via polycondensation of 1,5-pentanediamine and adipic acid by Cathay Industrial Biotech Ltd., Shanghai, China. Melt spinning was carried out at 275 °C, followed by a multistage drawing process to produce fibers. The spinning, thermal drawing, and annealing conditions were consistent with those reported in our previous work [13]. During the spinning process, the first godet roller speed was 180 m/min and the winding roller speed was 460 m/min, corresponding to an initial draw ratio of 2.55. In this study, we focused on an unexamined fiber orientation—along the fiber axis—using both ex situ and in situ datasets obtained under drawing speeds of 38 mm/min and 57 mm/min. In addition, unoriented film samples were prepared from the same nylon 5,6 polymer to support structural analysis.

### 2.2. Film Preparation and X-Ray Characterization

To investigate the properties of the γ-phase in its unoriented state, we prepared film samples using the same nylon 5,6 polymer. A 150 μm-thick copper foil was used as a mask to define the sample area, and hot pressing was conducted at 285 °C for 10 min. The molded films were demolded at room temperature and analyzed using synchrotron X-ray diffraction (XRD) at beamline 5A of the Pohang Accelerator Laboratory (PAL), Pohang, Korea. The wavelength of the incident X-rays was λ = 1.0716 Å (11.57 keV). These film samples were used to analyze the overall γ-phase structure and calculate the unit cell parameters.

### 2.3. Fiber Drawing and X-Ray Analysis

Tensile tests on the fiber samples were conducted at 90 °C under drawing speeds of 38 mm/min and 57 mm/min. This temperature was selected because nylon 5,6 has a reported glass transition temperature (Tg) of ~55 °C [8], and drawing is generally optimized at 20–30 °C above Tg, making 90 °C the most suitable condition for stable deformation without premature fiber breakage. For ex situ measurements, the samples were analyzed at room temperature after stretching. In contrast, in situ measurements were performed during stretching using a custom-designed uniaxial tensile stage equipped with a 3 mm-diameter X-ray window.

The analysis focused specifically on the (004) diffraction plane corresponding to the polymer chain axis (c-axis). Structural evolution was tracked based on the (004) peak, which is common to both α- and γ-phases. Diffraction planes observed perpendicular to the fiber axis, such as α(020), α(110), and γ(010), which were the focus of previous studies, were not considered here. The acquired 2D WAXS data were analyzed using the acquired 2D WAXS data were analyzed using the GISAXS package in Igor Pro (version 6.22A). Circular integration profiles through the (004) reflections were fitted in Origin with the Pearson VII function, and the phase fractions were quantified from the integrated peak areas in Q-space. At high draw ratios, the α(004) reflection could be clearly distinguished, and this information was used as the basis for determining phase fractions consistently across all conditions.

### 2.4. Computational Details

To calculate the detail structures of the α-phase and γ-phase of Nylon 5,6, density functional theory (DFT) simulations were performed using the Vienna Ab initio Simulation Package (VASP) [14,15,16]. The generalized gradient approximation (GGA) exchange–correlation functional of Perdew–Burke–Ernzerhof (PBE) [17] together with projector-augmented-wave (PAW) pseudopotentials [18] was employed for all calculations. An actual spacing of approximately 0.02 Å^−1^ was used to set the Monkhorst–Pack k-point mesh [19]. A plane-wave energy cutoff of 500 eV was employed, with convergence thresholds set to 1.0 × 10^−5^ eV for total energy and 2.0 × 10^−2^ eV/Å for atomic forces. To improve the accuracy of the DFT calculations, the Grimme DFT-D3 dispersion correction scheme [20] and dipole correction [21] along the z-direction were also incorporated. Full lattice relaxation was also conducted to optimize the lattice structures of the α-phase and γ-phase of Nylon 5,6.

### 2.5. Use of Generative AI Tools

Generative artificial intelligence (OpenAI’s ChatGPT, version 4o) was used in two distinct parts of this study: (1) to assist in re-constructing and visualizing initial atomic coordinate models for nylon 5,6 based on prior literature, which served as input structures for DFT calculations, and (2) to support the development of a custom Python script (Python version 3.13) for simulating powder X-ray diffraction patterns using experimentally refined unit cell parameters. All AI-assisted outputs were carefully reviewed, validated, and modified by the authors prior to use. The relevant coordinate models and scripts used in this study are not publicly available but can be obtained from the corresponding author upon reasonable request.

## 3. Results & Discussion

The initial as-spun nylon 5,6 fibers used in this study were produced under processing conditions of 8.47 g/min extrusion rate and a final take-up speed of 460 m/min. These conditions generally result in a semi-oriented state with relatively low crystallinity. However, X-ray diffraction measurements revealed that a well-defined γ-phase crystal structure was not present even under such low take-up speeds. This indicates that the as-spun fibers cannot be considered fully crystalline in the γ-phase, highlighting the need for a well-defined γ-phase reference sample to serve as a structural baseline for analyzing phase transformations during subsequent drawing. To this end, we fabricated unoriented film samples using the same nylon 5,6 material and conducted a quantitative analysis of their γ-phase crystal structure.

**Figure 1:**
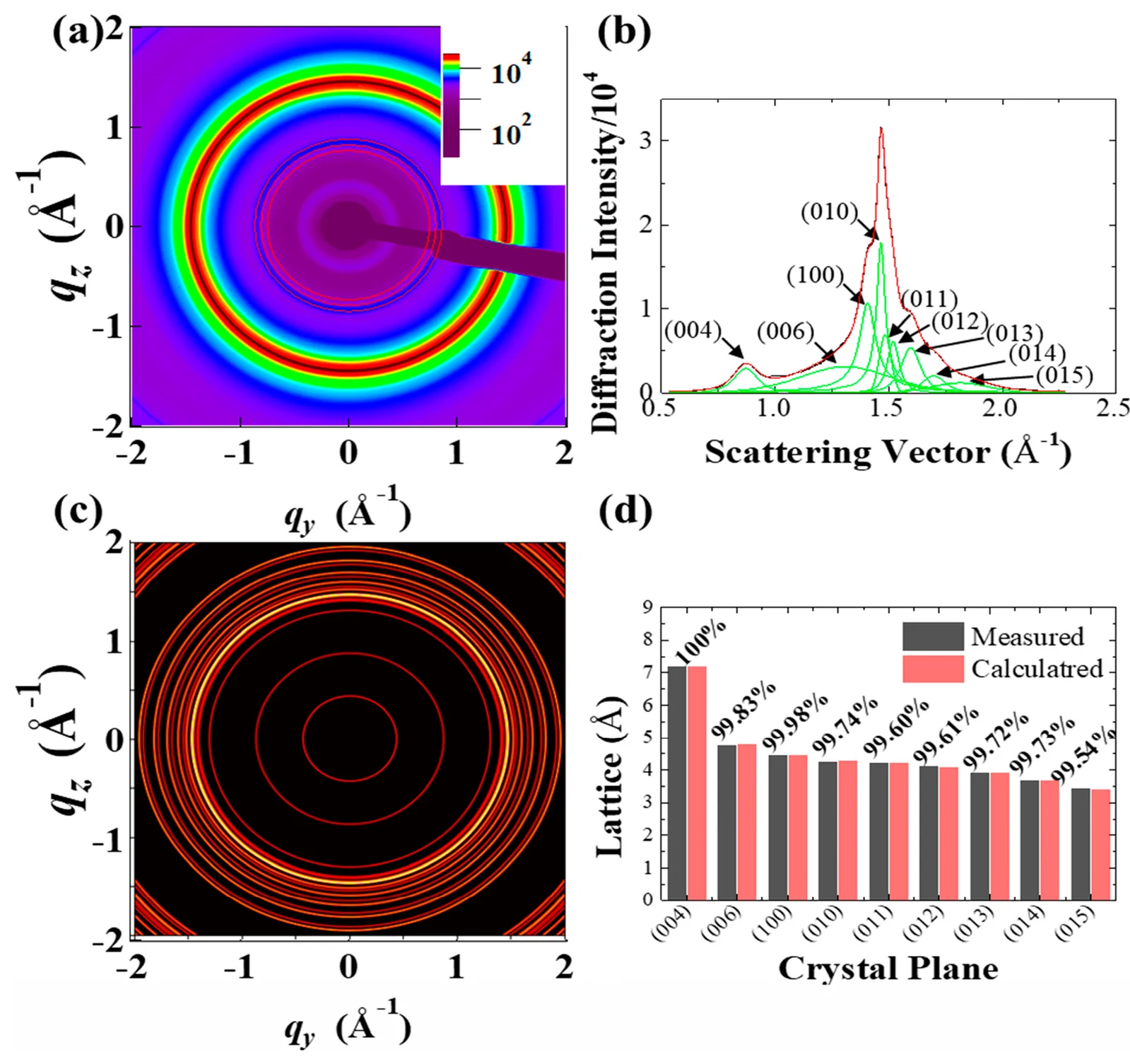
Structural characterization of the γ-phase in an unoriented nylon 5,6 film and validation of the refined unit cell model: (**a**) 2D WAXS pattern showing concentric rings indicative of isotropic crystallinity; (**b**) 1D diffraction profile extracted from (**a**), with nine γ-phase peaks fitted using Pearson VII functions; (**c**) simulated powder diffraction pattern based on refined unit cell parameters, showing excellent agreement with experimental data; (**d**) comparison of measured and calculated d-spacings, with all deviations within 0.1%, supporting the accuracy of the γ-phase model.

Figure 1a shows the two-dimensional WAXS pattern of the nylon 5,6 film measured in transmission mode. As expected, the unstretched film exhibits an isotropic ring pattern typical of a powder-like structure, confirming the absence of preferential chain orientation. This isotropic diffraction enables quantitative analysis of multiple crystal planes.

Figure 1b presents the one-dimensional diffraction profile extracted from Figure 1a, along with peak fitting results. A total of nine diffraction peaks were resolved and modeled using Pearson VII functions, from which precise d-spacings were derived. Each peak was assigned to a specific reflection of the γ-phase crystal structure, enabling detailed structural characterization of the film.

Using the extracted d-spacing values, we refined the unit cell parameters to best fit the experimental data, based on γ-phase lattice constants reported in the literature [10]. The ideal powder diffraction pattern calculated from the refined unit cell is shown in Figure 1c, and the excellent agreement in peak positions (matching >99%) confirms the high structural fidelity of our γ-phase model.

Figure 1d compares the experimentally determined d-spacings with those calculated from the refined unit cell. The detailed numerical values—experimental and calculated scattering vectors (q), the corresponding d-spacings, and the deviations (Δd) together with the relative errors (%)—are summarized in Table 1. All relative errors are within 1%, quantitatively validating the accuracy of the refined structural model. This analysis establishes a robust γ-phase reference, which provides a reliable baseline for interpreting phase transitions observed in drawn nylon 5,6 fibers.

**Figure 2:**
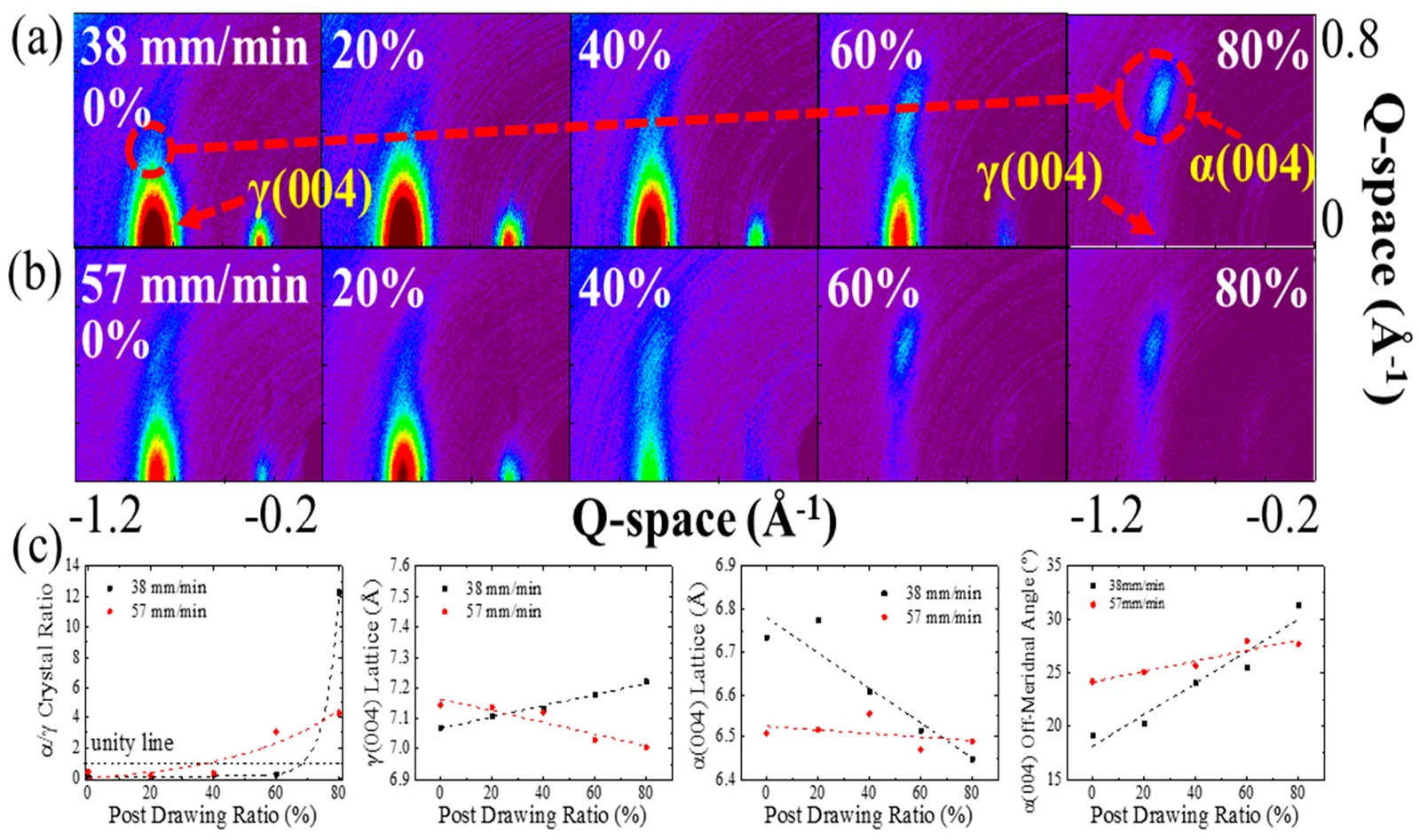
WAXS analysis of bio-based nylon 5,6 fibers stretched ex situ at two different drawing speeds, illustrating γ → α phase transition and structural evolution along the c-axis: (**a**) 2D WAXS patterns at various draw ratios under 38 mm/min drawing; (**b**) 2D WAXS patterns at various draw ratios under 57 mm/min drawing; (**c**) Quantitative results: (**left** to **right**) α/γ peak intensity ratio, γ(004) d-spacing, α(004) d-spacing, and off-meridional angle of α(004). The α/γ ratio was calculated from the integrated intensities of the α(004) and γ(004) reflections, obtained from the azimuthally integrated diffraction profiles.

Figure 2 presents the WAXS patterns and quantitative analyses of bio-based nylon 5,6 fibers subjected to ex situ stretching at two different drawing speeds (38 mm/min and 57 mm/min). Structural changes in the γ- and α-phases were examined based on the (004) diffraction plane, focusing on chain-axis alignment. In particular, the α/γ ratio shown in Figure 2c corresponds to the intensity ratio of the α(004) to γ(004) reflections, providing a quantitative measure of the relative phase fractions along the chain axis.

As shown in the WAXS images in Figure 2a,b, at 38 mm/min, the γ(004) reflection remained strong up to 60% draw ratio but abruptly disappeared at 80%, where a distinct α(004) peak emerged. In contrast, at 57 mm/min, the γ(004) signal began to decline from 40%, and α(004) appeared around 60%, though its intensity remained relatively weak thereafter. This suggests that although rapid drawing may trigger the phase transition earlier, it may lead to less complete crystal growth and chain ordering.

The quantitative analysis in Figure 2c confirms this trend. At 38 mm/min, the α/γ ratio remained below 1 (0.83, α-phase fraction 45.5%) up to 60% drawing, indicating a γ-phase dominant structure, but sharply increased to 12.3 at 80%, corresponding to an α-phase fraction of 92.5%. In contrast, at 57 mm/min the α/γ ratio showed a more gradual increase, reaching 4.29 at 80% drawing with an α-phase fraction of 81.1%, indicating a smoother transition. These behaviors were fitted using an exponential function (Equation (1)):

(1) $$y=A\cdot \exp\left(\frac{x}{t}\right)+y_{0},$$

Here, y denotes the crystalline ratio between the α- and γ-phases obtained from the integrated peak areas in Q-space; x is the applied post-drawing ratio (%) used as the strain variable; t is the fitting parameter that sets the characteristic strain scale of the transition (smaller t indicates a more rapid increase in the α/γ ratio with strain); A is the amplitude (scaling factor) of the exponential term; and *y*_0_ is a constant offset.

The resulting fitting parameter t was 4.36 for the slow drawing condition (38 mm/min), which is approximately one-seventh of that for the fast drawing condition (57 mm/min, t = 33.94). This indicates that the phase transition is significantly more sensitive to strain under slower drawing. The d-spacing of the γ(004) reflection also showed opposite trends depending on drawing speed. At 38 mm/min, it gradually increased with strain, likely reflecting a structural pre-transition toward the α-phase. In contrast, the d-spacing decreased at 57 mm/min, suggesting that under rapid stretching, the γ-phase rapidly collapsed before the pre-transitional state could be sufficiently captured.

Additionally, the off-meridional angle of the α(004) reflection indicates how far the α-phase crystals deviate from the fiber axis and is directly related to the β-angle of the monoclinic unit cell. Based on this relationship, the unit cell parameter *c* was quantitatively calculated using the following equation:

(2) $$c=\frac{4\cdot d_{004}}{\sin\left(\theta +90^{{}^{\circ}}\right)}$$

This equation enabled the assessment of structural evolution along the chain direction (c-axis) under different drawing conditions. The initial *c* values were 28.0 Å for 38 mm/min and 28.5 Å for 57 mm/min, increasing to 30.2 Å and 29.3 Å at 80% drawing, respectively. These results indicate that greater chain-axis extension occurred under slower drawing, accompanied by enhanced alignment. These findings indicate that slow drawing allows sufficient time for polymer chains to align and organize into the α-phase, enabling both nucleation and growth. In contrast, fast drawing may induce the phase transition but limit the extent of structural development. This demonstrates that drawing speed plays a critical role not only in initiating the γ-to-α transition but also in determining the degree of crystalline ordering achieved.

**Figure 3:**
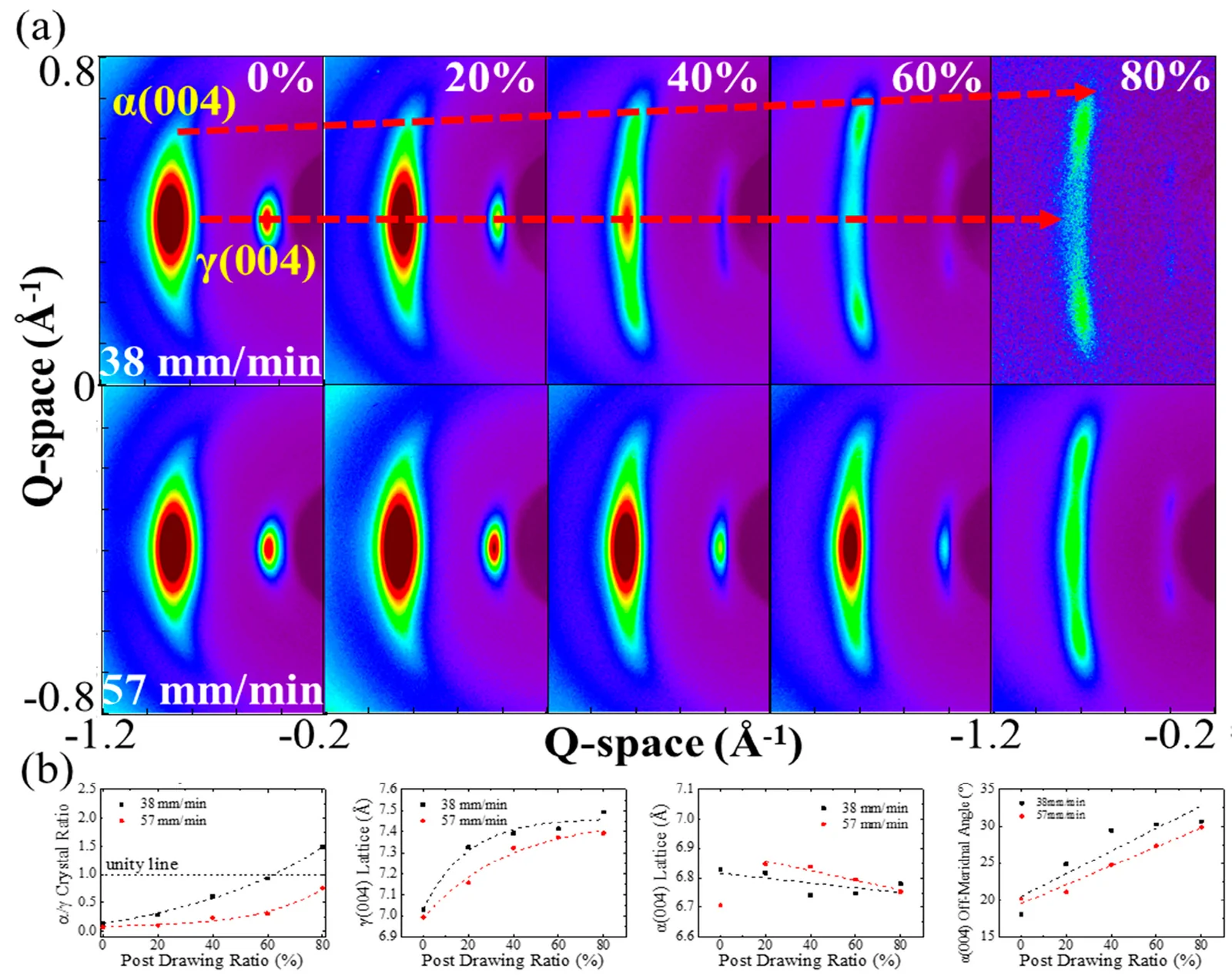
Structural evolution of bio-based nylon 5,6 fibers during in situ drawing at 90 °C: (**a**) 2D WAXS patterns captured at various drawing ratios under two different drawing speeds: 38 mm/min (**top**) and 57 mm/min (**bottom**). The γ(004) and α(004) reflections are indicated by dashed lines; (**b**) Quantitative analysis of phase and lattice parameters as a function of post-drawing ratio: (**left** to **right**) α/γ crystalline ratio, γ(004) d-spacing, α(004) d-spacing, and off-meridional angle of α(004).

Figure 3 presents in situ WAXS results obtained during uniaxial drawing at 90 °C, capturing real-time structural evolution of bio-based nylon 5,6 fibers. All data were acquired under constant temperature, ensuring that the observed structural changes directly reflect the effect of mechanical drawing, without thermal fluctuations.

As drawing progressed, the γ(004) reflection gradually weakened, but unlike in the ex situ experiments, it remained clearly visible even at the later stages of deformation. Notably, at 80% strain, the γ-phase was still dominant, and the α/γ crystalline ratio remained low. For 38 mm/min, the ratio was 0.93 at 60% strain (α-phase fraction 48.2%) and increased only to 1.49 at 80% (59.9%). For 57 mm/min, the α/γ ratio remained below 1.0 throughout, reaching only 0.76 at 80% strain with an α-phase fraction of 43.1%. This indicates that the γ-to-α structural transition proceeds much more slowly under in situ conditions compared to ex situ drawing.

The evolution of the γ(004) lattice spacing under in situ conditions exhibited a steady increase with strain for both drawing speeds, reflecting a typical lattice elongation behavior. As the strain approached 80%, this increase tended to plateau, suggesting saturation of molecular alignment along the chain direction (c-axis). This trend contrasts sharply with the non-monotonic γ(004) behavior observed under ex situ conditions in Figure 2, implying that the latter includes additional effects such as thermal shrinkage and structural relaxation after drawing.

In contrast, the α(004) lattice spacing under in situ conditions showed a slight decrease with increasing strain, regardless of the drawing speed. This behavior, unaffected by post-drawing thermal treatment or cooling, reflects pure structural changes during deformation. The initially high α(004) spacing observed at 0% strain under 57 mm/min is attributed to limited signal accuracy, as the α-phase fraction was only 6.8% and heavily overlapped with the γ-phase reflections. Overall, both speed conditions showed a consistent trend of decreasing α(004) d-spacing, which can be interpreted as stress relaxation and molecular rearrangement within the α-phase during drawing.

Meanwhile, the off-meridional angle of the α(004) reflection gradually increased with strain, paralleling the decrease in α(004) d-spacing. Based on these values, the unit cell parameter *c* was calculated using Equation (2). The c-axis length increased from 28.7 Å to 31.5 Å for 38 mm/min, and from 28.6 Å to 31.1 Å for 57 mm/min, confirming progressive alignment of polymer chains along the fiber axis and structural development within the α-phase.

Unlike conventional equatorial analyses of polymer fibers, where the Crystal Perfection Index (CPI) is often applied to deconvolute crystalline and amorphous contributions [22,23], our meridional diffraction profiles did not show a distinct amorphous halo. This can be attributed to the processing conditions: even the as-spun fibers had already experienced an initial draw ratio of 2.55 during spinning, which promoted considerable γ-phase crystallization and reduced the amorphous signal. Accordingly, the structural evolution captured in this study is more appropriately described as a γ → α phase transition rather than crystallization from an amorphous state. Furthermore, our analysis focuses on the diffraction along the fiber axis, a direction that has been less frequently examined in previous studies compared to the conventional equatorial profiles. By using the (004) reflections in this orientation, the present work highlights the phase transition pathway from a chain-axis perspective, which provides important complementary insight into the structural evolution of odd–even nylons.

These findings suggest that the γ-to-α phase transition in nylon 5,6 fibers is not completed during the drawing process itself, but rather initiated through orientation and nucleation triggers. The full structural transformation and crystal growth likely proceed after drawing, once the mechanical stress is released. In other words, the structure observed during drawing represents a preparatory or partially transformed state, while the completion of phase transition depends strongly on post-drawing relaxation. This supports a two-step transition mechanism in which molecular alignment and phase transition are temporally decoupled in fiber drawing processes.

**Figure 4:**
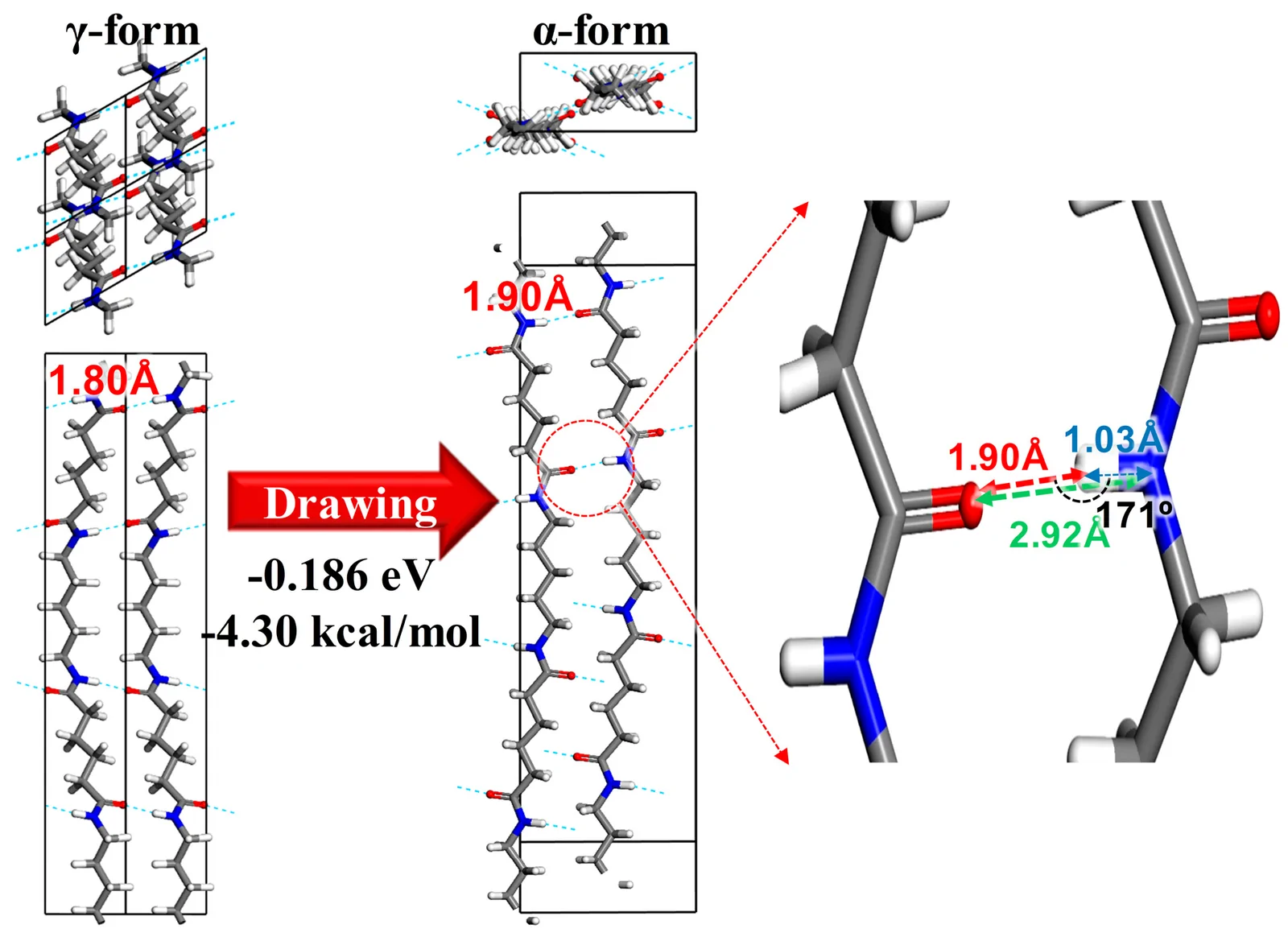
DFT-optimized unit cell structures of nylon 5,6 in the γ-phase (left) and α-phase (right) used to compare relative structural stability under drawing. Green dotted lines indicate intermolecular O···H hydrogen bonds. The inset highlights the hydrogen-bonding region of the α-phase structure, where green dotted lines represent interchain N···O distances, red dotted lines correspond to interchain O···H hydrogen bond lengths, blue solid lines indicate intrachain N–H covalent bonds, and black dashed arcs denote N–H···O bond angles. The γ-phase exhibits a twisted chain configuration with hydrogen bonds of 1.80 Å, whereas the α-phase exhibits a near-trans methylene backbone with hydrogen bonds of 1.90 Å length and a 171° bond angle, forming a more ordered network. The α-phase’s lower energy by ~4.3 kcal/mol suggests its thermodynamic preference under drawing-induced alignment.

Figure 4 presents a comparison of the relative stability between the γ- and α-phases of nylon 5,6, based on unit cell structures proposed in previous studies, using density functional theory (DFT) calculations. As shown in Table 2, the calculated lattice parameters closely match the reported reference values, confirming the accuracy of the structural modeling. Although the calculated potential energies fall within the range reported in the literature, some numerical differences may arise due to the use of updated functionals and basis sets. Nevertheless, the conclusion regarding the relative stability of the two phases remains consistent.

The calculated energy difference is approximately 4.3 kcal/mol, indicating that the α-phase is thermodynamically more stable than the γ-phase. Despite both phases exhibiting stable hydrogen bonding between adjacent chains, the lower energy of the α-phase suggests that ideal chain packing—such as extended trans conformations and linear chain alignment—plays a more critical role in determining phase stability than hydrogen bonding alone.

In particular, the α-phase exhibits a near-trans methylene backbone, except for slight deviations near the α-carbon adjacent to the amide group. The resulting hydrogen bonds are highly linear, with a bond length of ~1.90 Å and a bond angle of ~171°, forming a well-aligned hydrogen bonding network. In contrast, the γ-phase shows a shorter hydrogen bond length (~1.80 Å), but its twisted chain configuration results in a less linear hydrogen bonding arrangement.

Puiggalí et al. reported that distortions in bond angles and the topology of the hydrogen-bonding network are also critical factors in determining phase stability [6]. In odd-methylene nylons, certain carbonyl groups form hydrogen bonds with two amide NH groups in different directions, giving rise to a bifurcated hydrogen-bonding network. Unlike an ideal linear arrangement, this topology alleviates the geometric constraints imposed by the odd-numbered methylene sequence and thereby contributes to lattice stabilization. Our DFT calculations were performed on the two structural models of nylon 5,6 proposed in this earlier work and are in good agreement with the reported geometries. From this perspective, the observed γ → α transition in our study can be interpreted as being stabilized not only by differences in hydrogen-bond length and linearity but also by the combined effects of bond angle distortions and hydrogen-bond network topology.

These structural differences provide a fundamental explanation for the energetically favorable transition from γ- to α-phase under the application of external energy such as drawing. Although the γ-phase is stable at ambient conditions, the alignment of polymer chains during drawing facilitates a phase transformation into the more stable α-phase. The experimentally observed γ → α transformation behavior in this study is in good qualitative agreement with these computational findings.

This structural evolution also has direct implications for the macroscopic properties of nylon 5,6 fibers. Our previous ex situ tensile tests demonstrated that fibers drawn at lower speeds (e.g., 37 mm/min) exhibited slightly higher tenacity than those drawn at higher speeds (57 mm/min), and the difference became more pronounced at higher draw ratios [13]. In addition, density-gradient measurements under different draw ratios confirmed an increase in density with drawing [24]. Such density increases can be primarily attributed to the crystallization of amorphous regions, which enhances the overall crystallinity of the fiber. At the same time, the α-phase (α-like structure in odd-methylene nylons) itself possesses a higher intrinsic crystal density than the γ-phase. Considering that one repeating chain unit corresponds to the chemical formula C_22_H_22_O_4_N_4_ with a molar mass of 406.44 g mol^−1^, the theoretical crystal densities can be estimated from the unit cell parameters: 1.168 g cm^−3^ for the γ-phase (577.90 Å^3^, one chain per cell) and 1.274 g cm^−3^ for the α-phase (1059.26 Å^3^, two chains per cell). The combined effects of increased crystallinity and the stabilization of the intrinsically denser α-like phase provide the mechanistic basis for the observed improvements in tenacity and density of nylon 5,6 fibers.

## 4. Conclusions

This study systematically investigated the structural transformation from γ-phase to α-phase in bio-based nylon 5,6 films and fibers using in situ WAXS analysis and DFT calculations. The initial film exhibited a typical γ-phase crystalline structure, whereas the as-spun fiber showed partial coexistence of α-phase, implying that structural ordering had already initiated during the spinning and winding process.

Ex situ stretching experiments revealed that the transformation pathway and the extent of phase transition were strongly dependent on strain rate. At a slower stretching speed (38 mm/min), the α-phase emerged abruptly at the final strain stage with significant changes in lattice parameters and orientation angles, while faster stretching (57 mm/min) triggered an earlier transformation with less complete structural ordering. In contrast, in situ experiments at 90 °C revealed a much slower γ → α transition, suggesting that full structural conversion occurs more effectively in the relaxed state after elongation.

DFT calculations confirmed that the α-phase is energetically more stable than the γ-phase by approximately 4.3 kcal/mol. The energy difference is attributed not to the presence or absence of hydrogen bonding—since both phases retain full hydrogen bonding—but rather to differences in molecular chain arrangement. Specifically, the α-phase adopts a predominantly near-trans conformation, except in the vicinity of the amide groups, resulting in greater linearity and lower energy.

These findings collectively provide new insight into the thermodynamic and structural basis of the γ → α transition in nylon 5,6. The combination of in situ synchrotron WAXS experiments and complementary DFT calculations demonstrates a consistent picture of the transition pathway. Importantly, this γ → α transformation also underlies the observed improvements in fiber tenacity and density, reflecting both enhanced crystallization of amorphous regions and the intrinsically higher crystal density of the α-like phase. Overall, this study provides a comprehensive understanding of nylon 5,6 by integrating structural evolution, theoretical analysis, and macroscopic properties.

## Figures and Tables

**Table 1:** Comparison of experimental and calculated diffraction peak positions for the γ-form crystalline phase obtained from nylon 5,6 films. The table lists experimental scattering vectors (q) and cor-responding d-spacings, calculated values derived from the refined unit cell, and their deviations (Δd) and relative errors (%). The small deviations (<1.0%) confirm the high consistency between the measured data and the structural model.

Reflection	q_exp_ (Å^−1^)	d_exp_ (Å)	q_cal_ (Å^−1^)	d_cal_ (Å)	Δd (Å)	Error (%)
**(002)**	0.436	14.400	0.436	14.424	0.024	0.2
**(004)**	0.871	7.212	0.871	7.212	0.000	0.0
**(006)**	1.309	4.800	1.307	4.808	0.008	0.2
**(100)**	1.407	4.465	1.407	4.466	0.000	0.0
**(010)**	1.465	4.287	1.462	4.298	0.011	0.3
**(011)**	1.484	4.234	1.478	4.251	0.017	0.4
**(012)**	1.520	4.135	1.525	4.119	−0.016	−0.4
**(013)**	1.597	3.935	1.601	3.924	−0.011	−0.3
**(014)**	1.697	3.702	1.702	3.692	−0.010	−0.3
**(015)**	1.814	3.463	1.823	3.447	−0.016	−0.5

**Table 2:** DFT-optimized lattice parameters of nylon 5,6 α- and γ-phases show good agreement with reference values, confirming the accuracy of the structural models.

		Lattice					
		a (Å)	b (Å)	c (Å)	α (°)	β (°)	γ (°)
**Alpha**	**Ref**	5.12	8.64	31.457	90	125.7	90
	**DFT**	5.259	8.613	32.133	90.18	133.30	89.95
	**Error (%)**	2.7	−0.3	2.1	0.2	6.0	−0.1
**Gamma**	**Ref**	4.88	4.79	28.8	90	90	59.3
	**DFT**	4.812	4.761	29.252	90.04	90.01	59.58
	**Error (%)**	−1.4	−0.6	1.6	0.0	0.0	0.5

## Data Availability

The raw data supporting the conclusions of this article will be made available by the authors on request.
